# Brain drain of healthcare professionals from Pakistan from 1971 to 2022: Evidence-based analysis

**DOI:** 10.12669/pjms.39.4.7853

**Published:** 2023

**Authors:** Sultan Ayoub Meo, Tehreem Sultan

**Affiliations:** 1 Sultan Ayoub Meo, MD, PhD, FRCP Department of Physiology, College of Medicine, King Saud University, Riyadh, 11461, Saudi Arabia.; 2 Tehreem Sultan, Harvard Law School, Harvard University, Massachusetts, USA

**Keywords:** Brain drain, Healthcare professionals, Intellectual migration, Pakistan

## Abstract

Since the creation of Pakistan in August 1947, political instability has been a persistent issue in the country, causing a migration of highly qualified, skilled people, and healthcare professionals. From 1971 to 2022 the total number of highly qualified and skilled people including healthcare professionals who migrated from the country is 60,19,888. Among them, 251677 (4.18%), were highly qualified, 455097 (7.55%) were highly skilled, and 5313114 (88.27%) were skilled professionals. Moreover, 50110 (0.83%) were healthcare professionals including doctors 31418 (62.69%), nurses 12853 (25.64%), and pharmacists 5839 (11.65%). The unsustainable political environment, lack of advanced technology-based institutes, poor healthcare infrastructure, low job opportunities and salary benefits in Pakistan caused the brain drain of highly qualified people including healthcare professionals. It adversely affected the academic institutes, the healthcare system, socio-economic growth, research productivity, and the development of the nation. The government of Pakistan must establish sustainable policies to minimize the brain drain of highly qualified people, and healthcare professionals, and recuperate the prosperity of their academic institutes and healthcare system for better healthcare services, and the advancement and sustainable development of the nation.

## INTRODUCTION

Pakistan is home to 231.4 million people,^1^ blessed with many rivers, mountains, minerals, natural gas reserves, coal and salt mines, and well-fertile agricultural land with multi-seasonal products. The country has 247 universities and degree-awarding institutions,^2^ including 176 medical and dental schools.^3^ Since the creation of Pakistan in August 1947, political instability has been a persistent issue in the country.^4^ Political instability reduces economic growth, threatens regional and foreign investors, and minimizes people’s savings, earning capacity and purchasing powers. Moreover, political instability causes inflation and unemployment, creating social unrest and uncertainty among people.^5^ An unstable political environment creates ambiguity among the public, academicians, healthcare workers, and researchers, and causes uncertainty in policies and decisions.^6^ The sociopolitical unrest significantly contributes to the instability in low and middle-income countries and causes a brain drain of skilled professionals,^7^ academicians, researchers, and healthcare professionals. The literature is lacking in highlighting the barn drain from Pakistan. This article emphasizes the brain drain of highly skilled people and healthcare professionals from Pakistan during the period 1971-2022.

### Brain Drain in Pakistan: 1971 to 2022:

**Fig.1:**
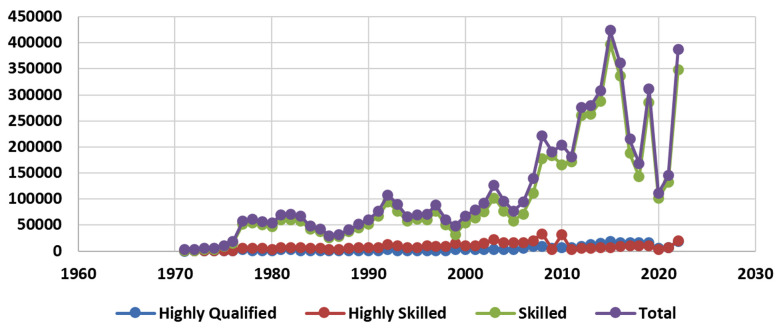
Migration of highly qualified, highly skilled people including healthcare Professionals from Pakistan (1971-2022).

**Fig.2:**
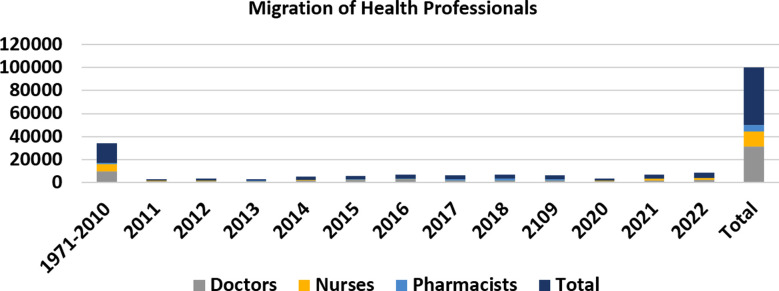
Migration of healthcare professionals from Pakistan during the period (1971-2022).

From 1971 to 2022 the total number of highly qualified and skilled professionals who migrated from Pakistan is 60,19,888. Among them, 251677 (4.18%), were highly qualified, 455097 (7.55%) were highly skilled, and 5314004 (88.27%) were skilled professionals (Table-I, Fig.1). While analyzing the profession of these highly qualified people, it was found that 50110 (0.83%) were healthcare professionals including doctors 31418 (62.69%), nurses 12853 (25.64%), and pharmacists 5839 (11.65%) (Table-II, Fig.2).

**Table-I:** Brain drains of highly qualified and skilled professionals including healthcare professionals from Pakistan (1971-2022).^8^

Year	Highly Qualified	Highly Skilled	Skilled	Total
1971	163	892	1499	2554
1972	782	904	1860	3546
1973	916	954	3408	5278
1974	954	582	3992	5528
1975	985	569	8848	10402
1976	835	1529	15087	17451
1977	2570	4413	51845	58828
1978	2155	5903	53805	61863
1979	1527	5245	49756	56528
1980	1729	4041	47569	53339
1981	2467	6984	60503	69954
1982	2190	7449	60748	70387
1983	2123	6473	58042	66638
1984	1427	4527	42005	47959
1985	968	4259	37244	42471
1986	717	3787	25225	29729
1987	796	3558	27294	31648
1988	743	4739	36276	41758
1989	925	6095	44483	51503
1990	1115	6834	52895	60844
1991	1308	7752	67215	76275
1992	2293	11653	93795	107741
1993	1908	10105	77820	89833
1994	1328	6916	58197	66441
1995	1292	7681	61177	70150
1996	1794	10168	59816	71778
1997	1669	9292	76599	87560
1998	2024	8230	50122	60376
1999	2699	13860	31678	48237
2000	2999	10292	54110	67401
2001	3155	10846	64098	78099
2002	2618	14778	74968	92364
2003	2719	22152	101713	126584
2004	3291	15557	77033	95881
2005	3737	15467	57793	76997
2006	5708	16332	71898	93938
2007	8178	20975	110938	140091
2008	9713	33173	177791	220677
2009	4954	3260	182657	190871
2010	7081	31650	165726	204457
2011	6974	3018	171672	181664
2012	9298	4202	261531	275031
2013	12057	5032	263138	280227
2014	14647	6216	287649	308512
2015	17484	7853	397317	422654
2016	16510	8172	335671	360353
2017	16029	9886	188745	214660
2018	16105	9770	142486	168361
2019	15525	9899	285960	311384
2020	5121	3745	102336	112092
2021	7396	6563	131348	145307
2022	17976	20865	347733	386574

Total	251677	455097	5313114	6019888

**Table-II:** Healthcare professionals migrated from Pakistan during the period 1971-2022.^8^

Healthcare Professionals	1971-2010	2011	2012	2013	2014	2105	2016	2017	2018	2019	2020	2021	2022	Total
Doctors	9854	1453	1218	1131	2074	2276	2779	1632	1945	1678	1223	1691	2464	31418
Nurses	6429	131	449	315	251	223	271	293	177	337	421	1788	1768	12853
Pharmacists	673	48	167	187	171	335	365	1217	1346	1121	67	66	76	5839

Total	16956	1632	1834	1633	2496	2834	3415	3142	3468	3136	1711	3545	4308	50110

While analyzing the data for the year 2022, about 832,339, skilled professionals headed abroad. Among them, 17976 (2.15%) were highly qualified and 20865 (2.50%) were highly skilled professionals. It shows that 2312 people left their homeland per day during the recent year. Among them, 2,464 (0.29%) were doctors, 1768 (0.21%) were nurses and paramedics (Table-I & II and Fig.1 & 2).

### Brain drain: origination and destination:

From 1971 to 2022, most people migrated from Pakistan to Saudi Arabia, UAE, Kuwait, Qatar, Oman, Bahrain, South Korea, Malaysia, the UK, USA, Switzerland, China, Brunei, and Germany. In the recent year 2022, the people travelled from Pakistan to the Saudi Arabia 514909 (61.86%), UAE 128477 (15.43%), Oman 82380 (9.89%), Malaysia 6175 (0.74%), Qatar 57999 (6.96%), Bahrain 3653 (0.43%), UK 2922 (0.35%), Cyprus 2906 (0.34%), Iraq 2387 (0.28%), Kuwait 2089 (0.25%), South Korea 2025 (0.24%), Japan 900 (0.10%), USA 801 (0.09%), China 673 (0.08%), Italy 350 (0.04%), and 23693 (2.845) people were left to the rest of the world.^8^

In the year 2022, people who migrated from Pakistan are from Islamabad 83169 (9.99%), Lahore 66708 (8.01%), Karachi 44341 (5.32%), Faisalabad 28385 (3.41%), Peshawar 20519 (2.46%), Rawalpindi 12437 (1.49%), Multan 7563 (0.90%), Abbottabad 6737 (0.80%), Jamshoro 5924 (0.71%), Bahawalpur 4788 (0.57%), Quetta 4328 (0.51%). These are the major cities of Pakistan from where most people migrated abroad.^8^

### Brain drain factors:

The brain drain or the human capital flight, occur in their pursuit of better living situations, high wages, advanced technology base environment, and better political conditions in various places worldwide. People pursue their careers because of the freedom of independence, and intellectual satisfaction of creativity.^9^ Although these characteristics are inspiring, society always needs minds of creative thinking. There are multiple factors including political instability influence the migration of skilled people from Pakistan. The most concerning factor is that young people are not the only ones who are rushing for the exit, people in their middle age are also trying to move out of the country due to unemployment, inflation, poverty, security, and economic issues.^9^,^10^

The people get disheartened because of low incentives for their academic credentials and experience causing them to migrate to developed countries. The common reasons why the brain drain takes place are fewer career options, low salary packages, lack of benefits, low quality of life, political instability, and crime conditions.^10^,^11^ Moreover, long term war in Afghanistan also effected the state and caused brain drain. The brain drain of highly qualified people including physicians, researchers and academicians adversely affected the academic institutes, science, research productivity, socioeconomic growth and sustainable development of the nation.^12^

### Impact of brain drain on academia and research:

In Pakistan, political instability, lack of job opportunities and limited resources negatively affect the progress and prospects of universities and academic institutions and cause the university faculty to flee from their universities and homeland.^11^ The science faculty not only migrate but also carry inventions and scientific prints. The migration of university faculty members developed a gap in the global standing of universities. This may be one of the reasons that Pakistani universities did not achieve a place among the top-ranked universities in the world.^13^,^14^ Although, Higher Education Commission (HEC) was established in year 2002, and a lot of efforts were made, opportunities were provided to enhance the quality of research by foreign collaborations, but the important aspect of brain drain was not amply addressed.

More recently, Nadir et al 2023^15^ reported that one in three medical students intends to migrate abroad after graduation due to a lack of resources and mismanagement in Pakistan. This has been adversely affecting Pakistan’s health system. Saluja and colleagues, 2020^16^ estimate the cost due to mortality linked with physician migration. The authors reported an annual loss of about $15·86 billion with the greatest costs incurred by India, Nigeria, Pakistan, and South Africa. The economic, social, and political instability in low-middle-income countries has induced further migration waves of healthcare workers compounding the pressure on already overstretched health systems.^16^

The recent wave of political instability in Pakistan in the year 2022 caused the migration of about 832,339 highly qualified and skilled people including healthcare professionals to head abroad. The migration of such a large number of professionals is likely to negatively impact research productivity and visibility. From January 2000 to December 2022, the number of articles published in the web of science-indexed journals worldwide was 248457. As per the Web of Science 2022 report, the rising trend decreased in the year 2022.^17^ The most potential reason for decreasing research productivity may be the political instability and brain drain from Pakistan.

In Pakistan, there are a total of 380 Higher Education Commission (HEC) indexed journals in various academic disciplines.^18^ Out of 380 HEC-indexed academic journals only 11 (2.89%) academic journals achieved a place in the Web of Science and quartile ranking. Among these journals only one journal, the Pakistan Journal of Medical Sciences (Impact Factor 2.340) crossed the IF of more than 2.0; the remaining journals have an impact factor of between 0.57-1.80.^17^ The highly qualified and skilled people are sending regular remittances, but it cannot compensate the loss of country in terms of qualified people that are much needed to participate in the universities, research institutes, and healthcare sector for the overall prosperity of the nation. It must be analyzed deep down whether this compensation is good enough or whether it is a great loss for the country to lose the highly qualified and skilled professionals who could help the country in a better way rather than just sending the remittances earned. The higher number of highly qualified and skilled professionals who departed the country is a cause of concern and it decreases academic and research productivity.

Science itself is one of the more migrant professions, and many scientists’ cross borders in search of better options and opportunities. Today, more people live outside the country of their birth than ever before.^19^ Knowledge and research productivity is a borderless enterprise, but some states such as Pakistan are worried that they are losing their top researchers. The worldwide highly cited scientists, one in eight scientists were born in developing countries, and 80% of those had since moved to developed states.^20^ A large number of Pakistan intellectuals try to return to their placental place after staying a long period in developed nations but once they return too late, they feel misfits in the system and their career structure. Moreover, the system is not easily accepting these intellectuals, hence the brain drain is a highly challenging issue for the state.

## CONCLUSIONS

Over the last fifty years, about six million highly qualified and skilled professionals migrated from the country. The unsustainable political environment, poor healthcare infrastructure, low job opportunities and salary benefits in Pakistan caused the brain drain of highly qualified people including healthcare professionals. Moreover, Afghanistan war and war on terror also had a compounding adverse affect on Pakistan’s state, society and brain drain. It adversely affected the academic institutes, healthcare system, socio-economic growth, research productivity, and the development of the nation. The government of Pakistan must establish sustainable policies to minimize the brain drain and recuperate the prosperity of their academic institutes and healthcare system for better healthcare services, and the advancement and sustainable development of the nation.

### Authors’ Contributions:

**SAM:** Study design, writing and editing the manuscript.

**TS:** Literature review, data collection, entry, and checking and analysis.
